# Supplementary material for: phylotaR: An Automated Pipeline for Retrieving Orthologous DNA Sequences from GenBank in R
Source: Life (Basel). 2018 Jun 5;8(2):20. doi: 10.3390/life8020020 (PMC6027284; doi:10.3390/life8020020)
Supplement: Supplementary file 1 [file life-08-00020-s001.zip › figure_S2.pdf]

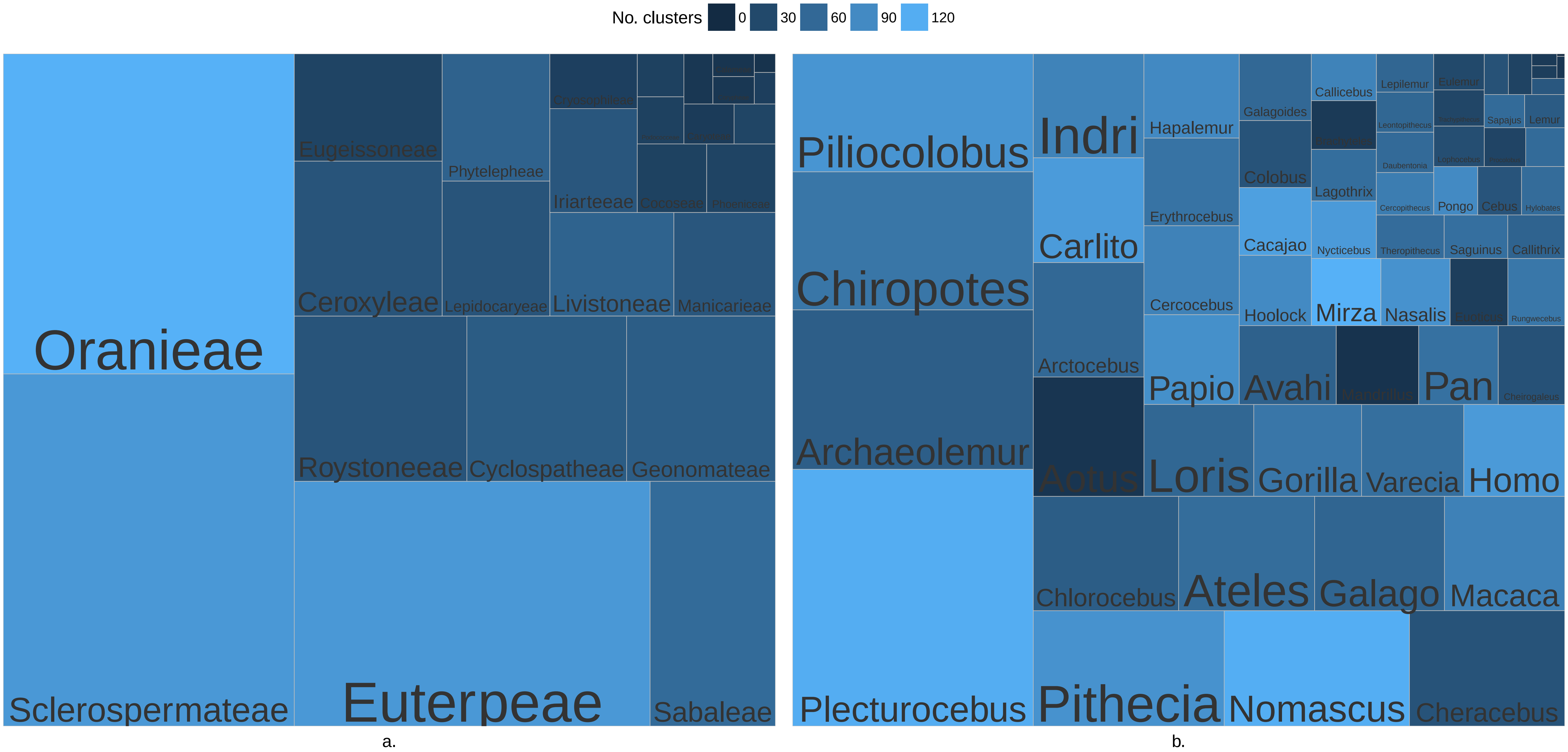

Figure S2. Relative distribution of number of sequences (box size) and clusters (colour) across tribes/genera for palms (a) and primates (b).
