# Supplementary figures and images for: phylotaR: An Automated Pipeline for Retrieving Orthologous DNA Sequences from GenBank in R
Source: Life (Basel). 2018 Jun 5;8(2):20. doi: 10.3390/life8020020 (PMC6027284; doi:10.3390/life8020020)

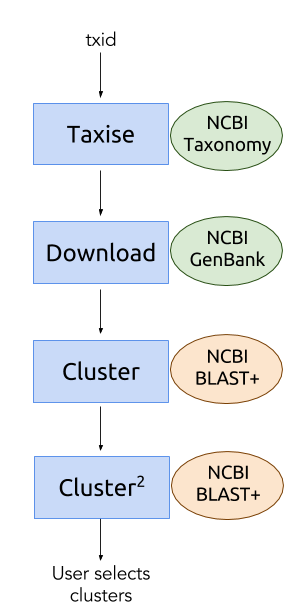

Supplement: Supplementary file 1 [file life-08-00020-s001.zip › figure_S1.png]

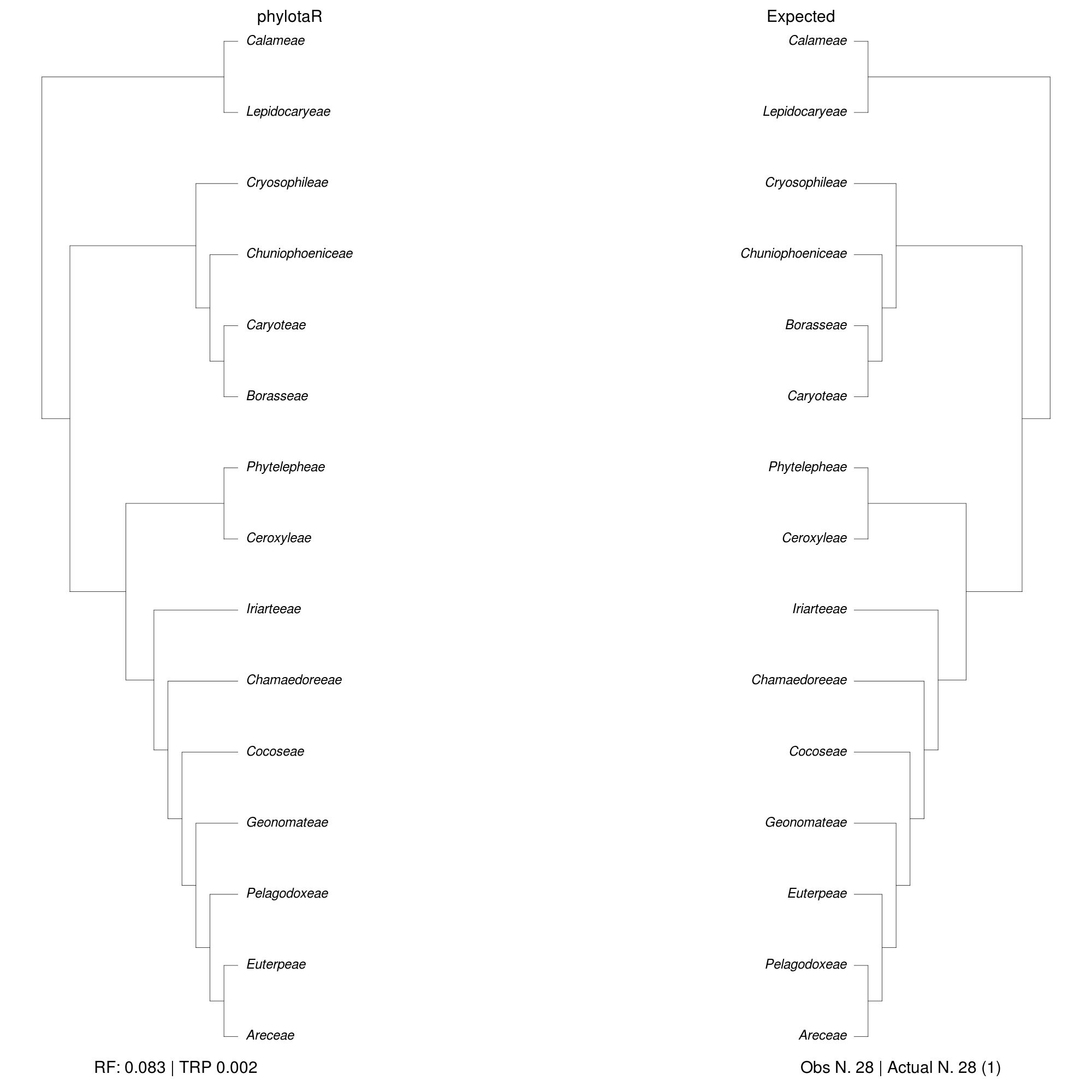

Supplement: Supplementary file 1 [file life-08-00020-s001.zip › figure_S4.png]

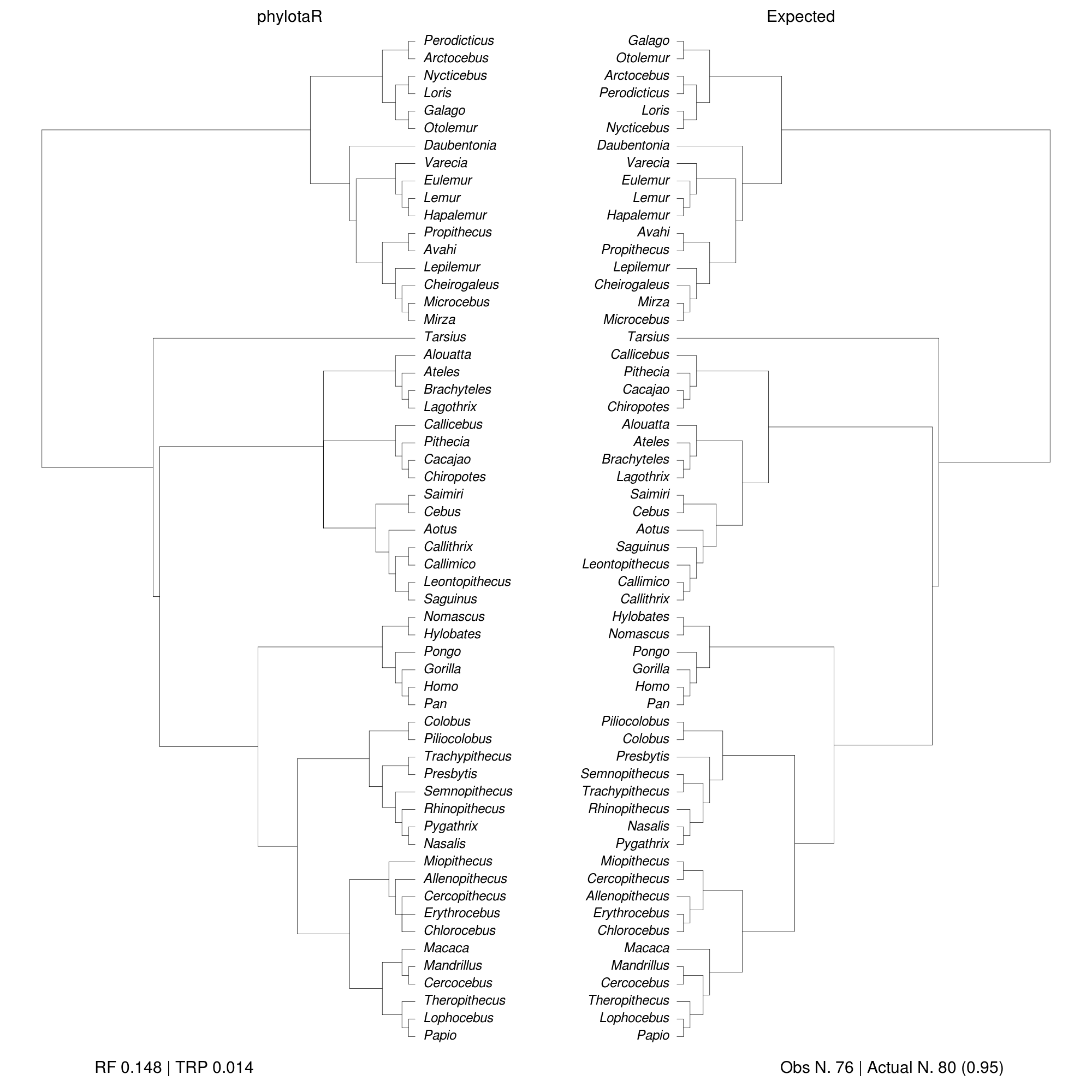

Supplement: Supplementary file 1 [file life-08-00020-s001.zip › figure_S5.png]
